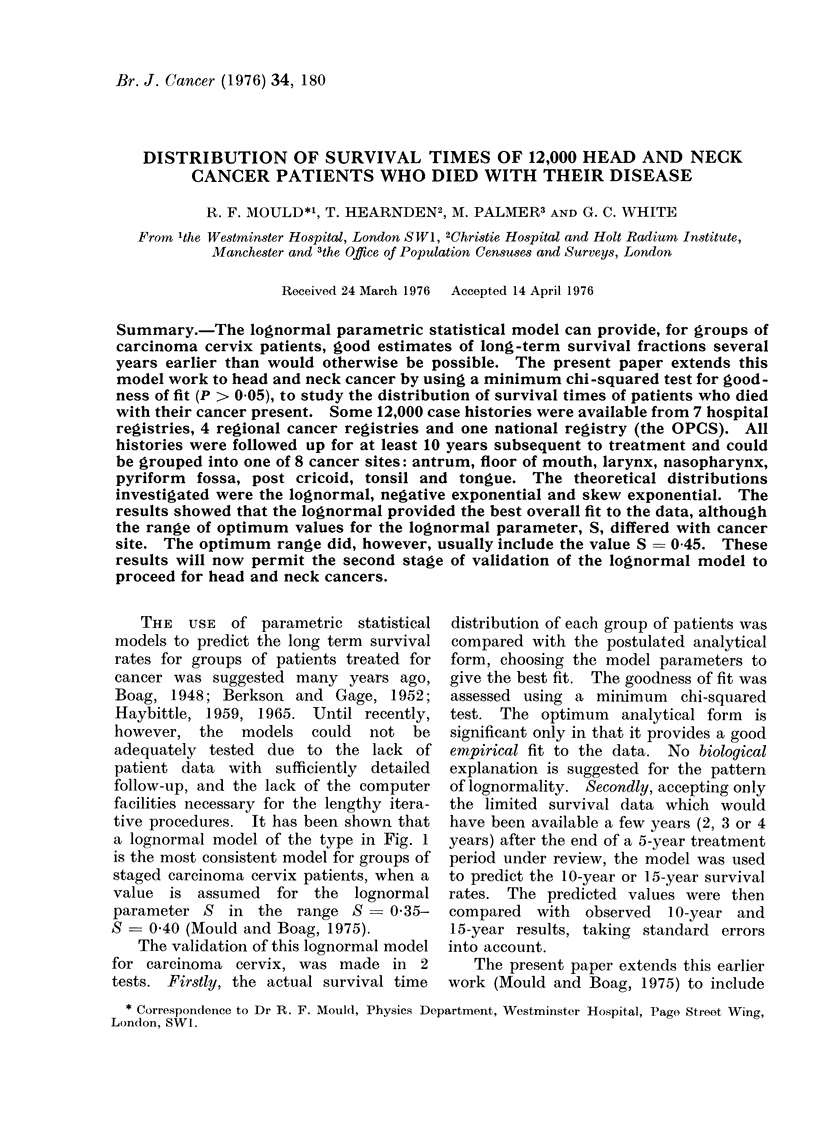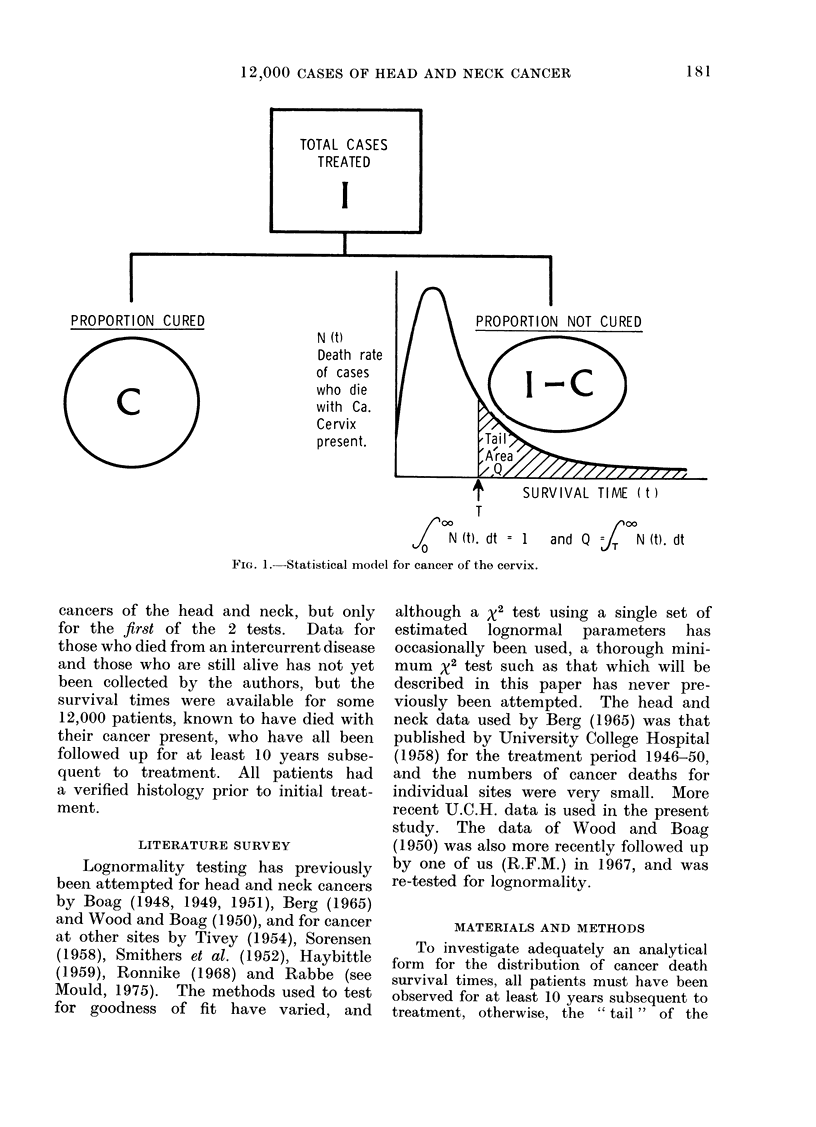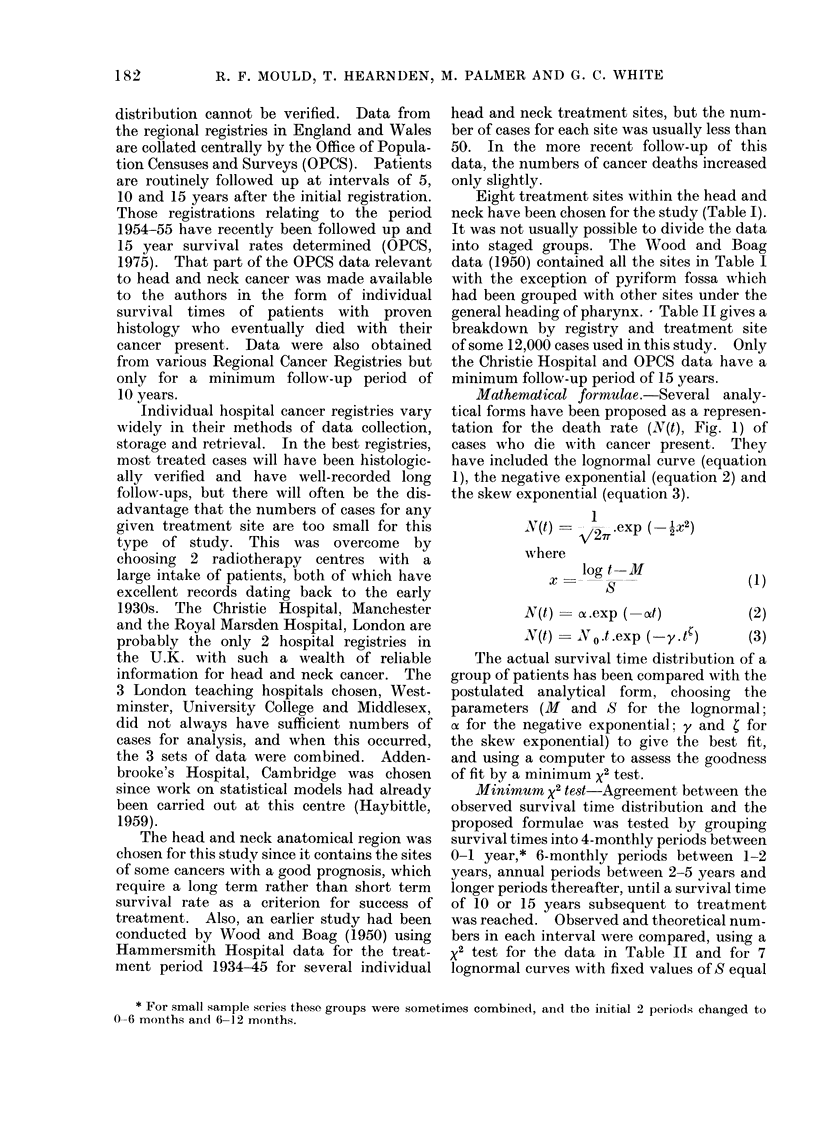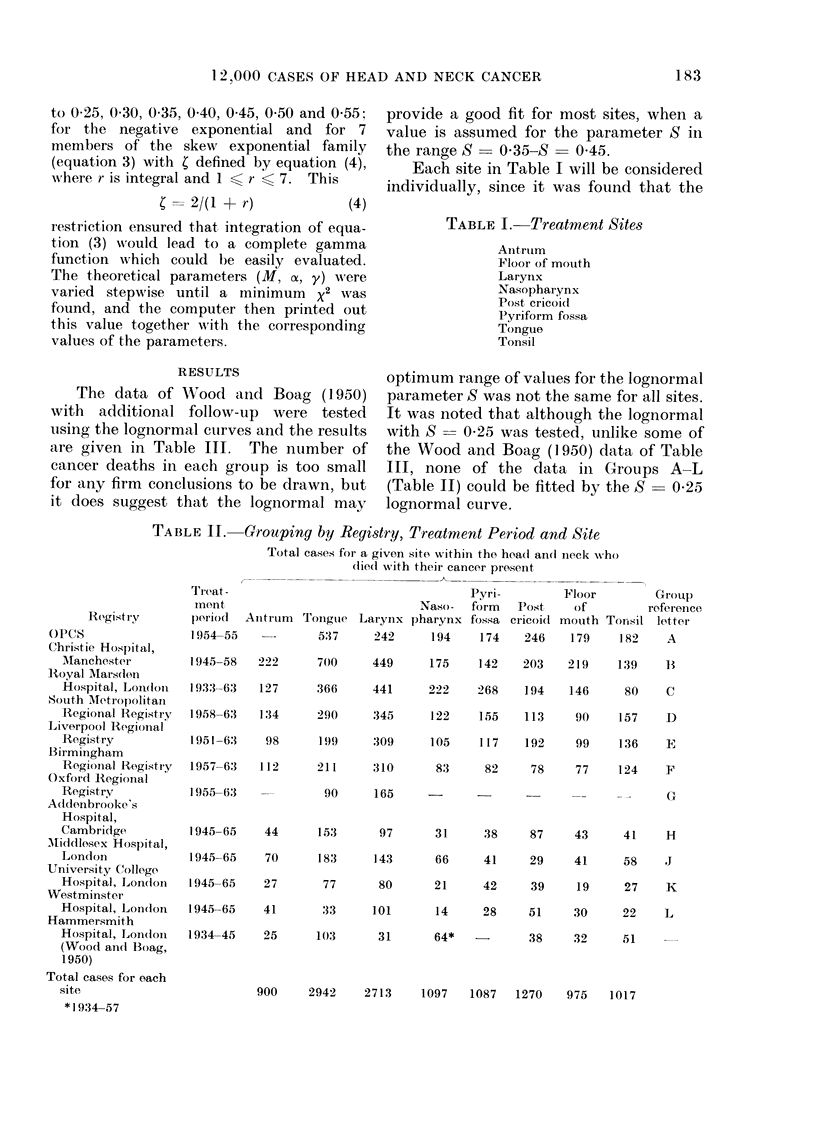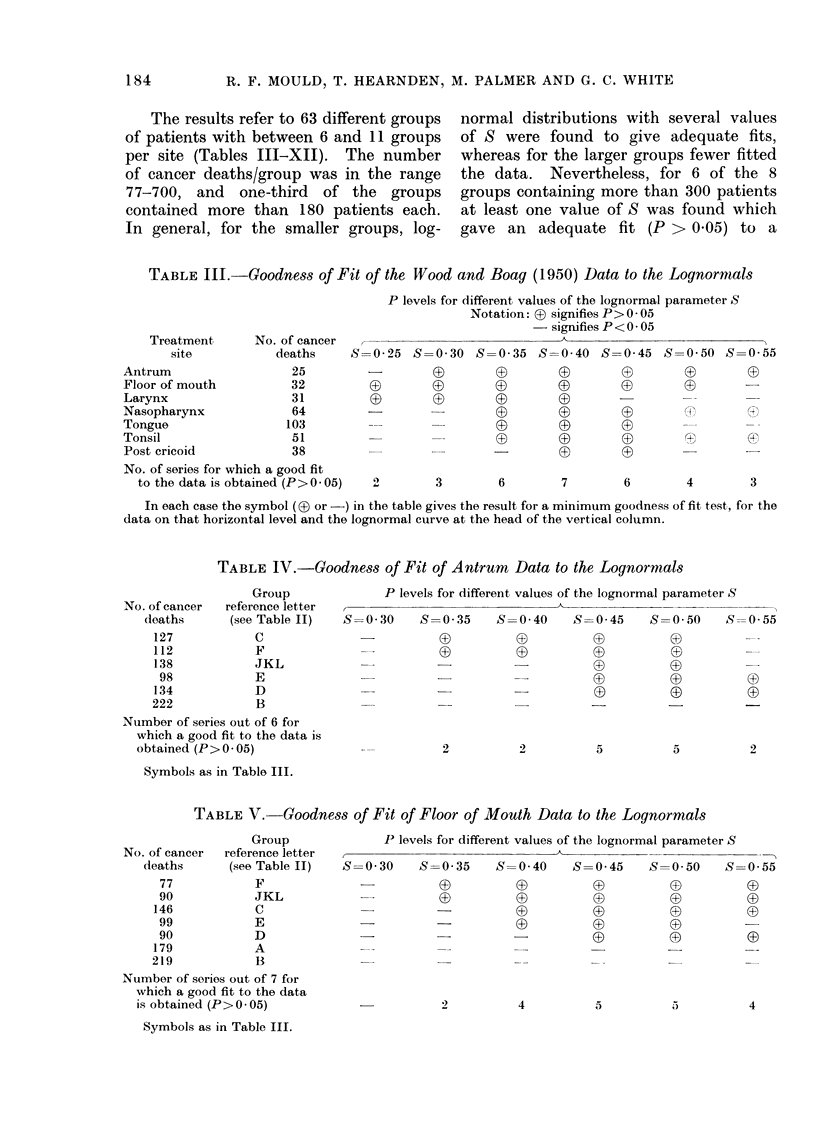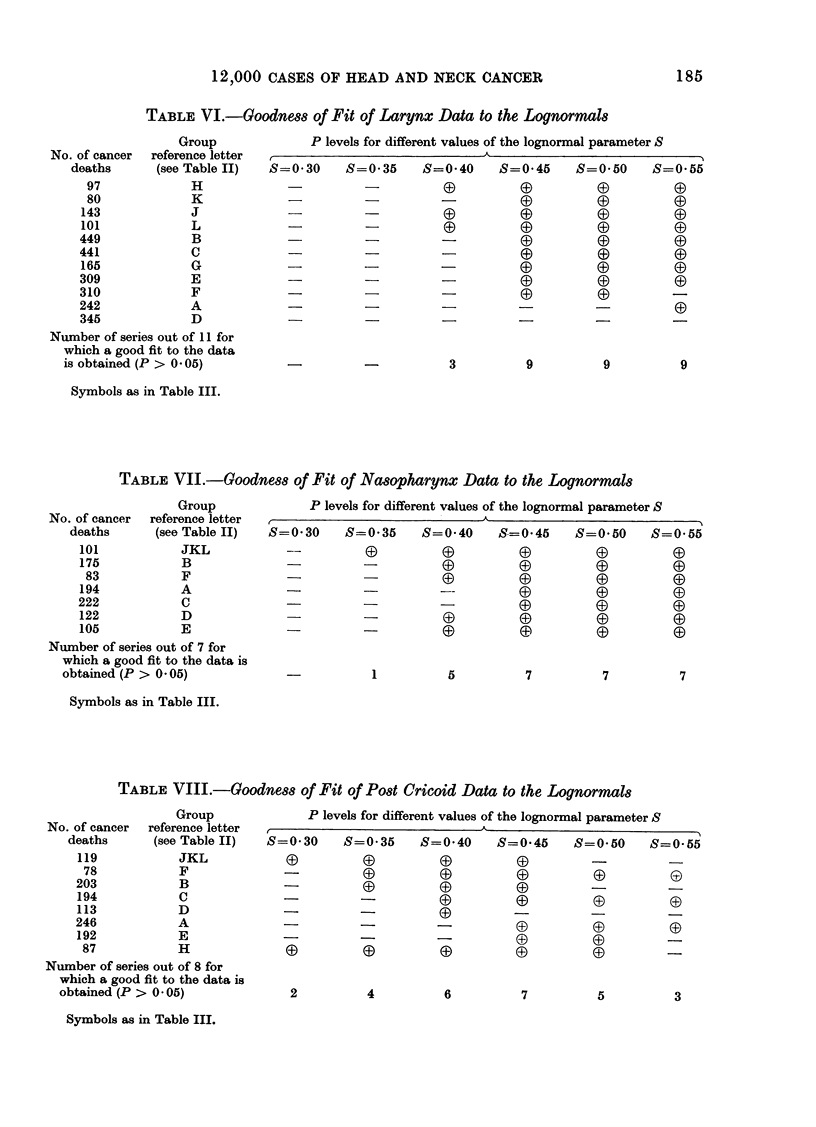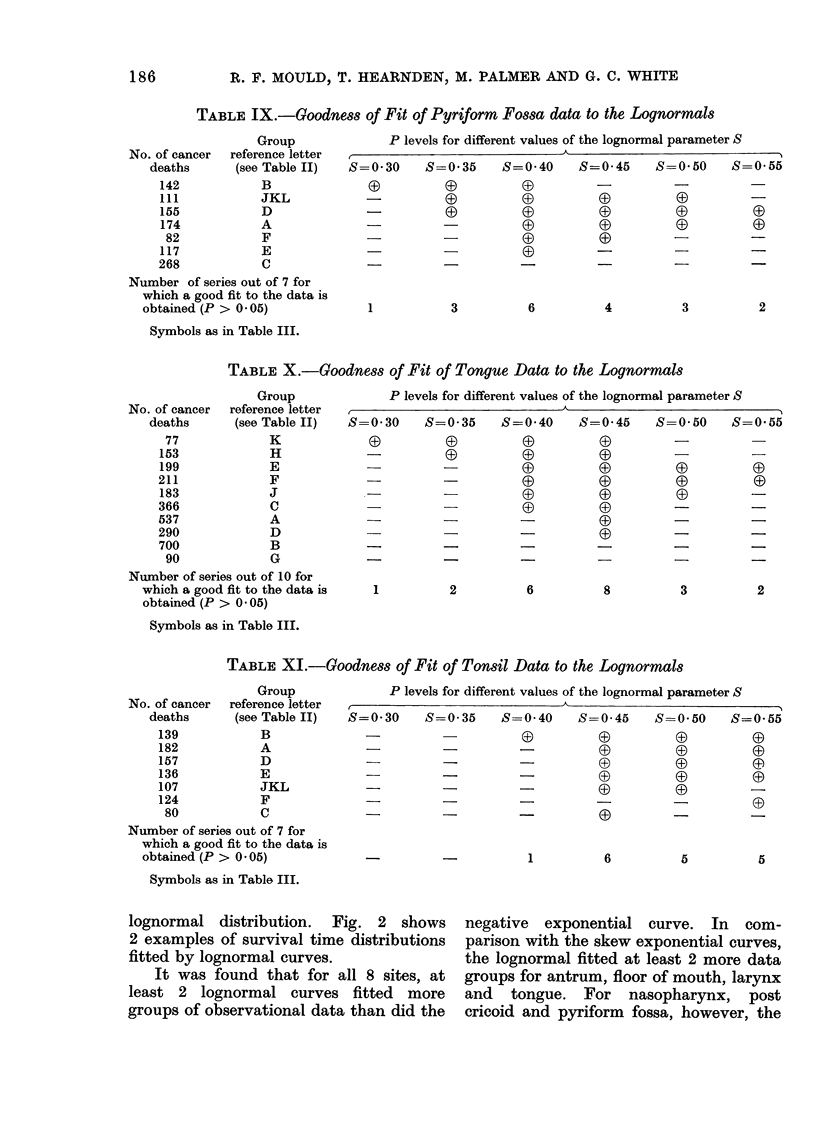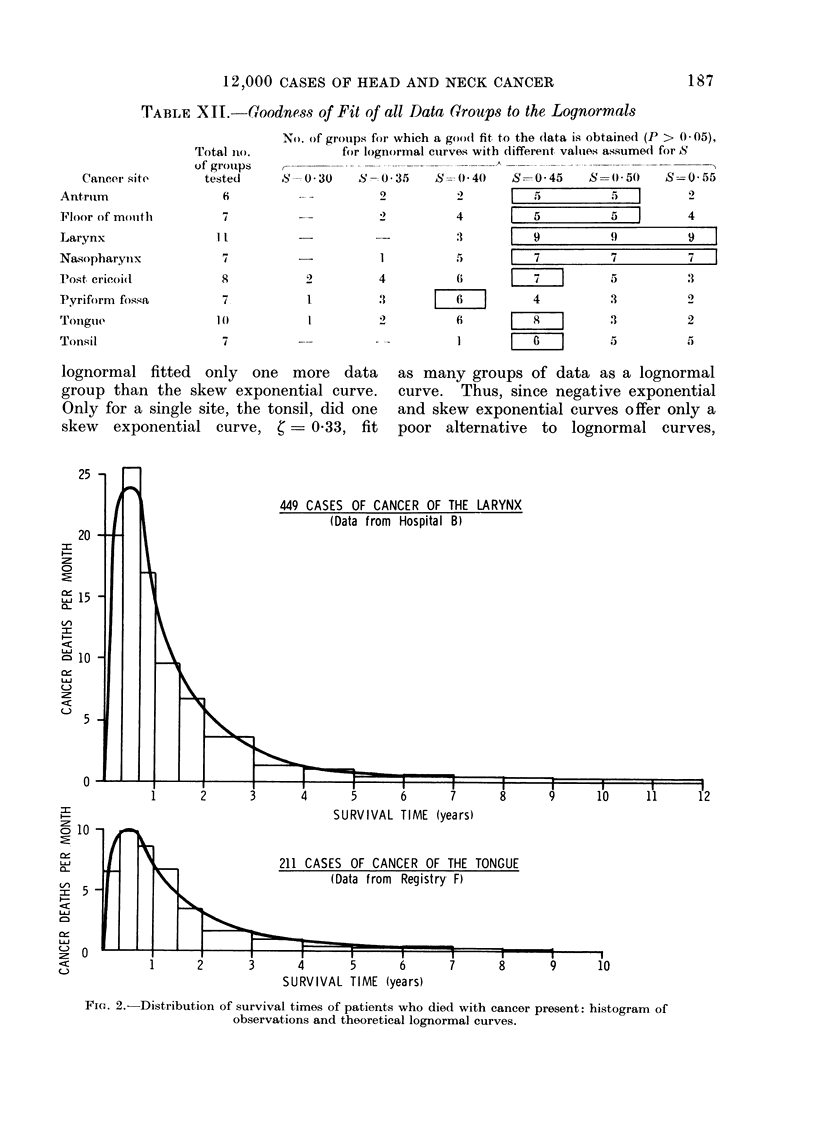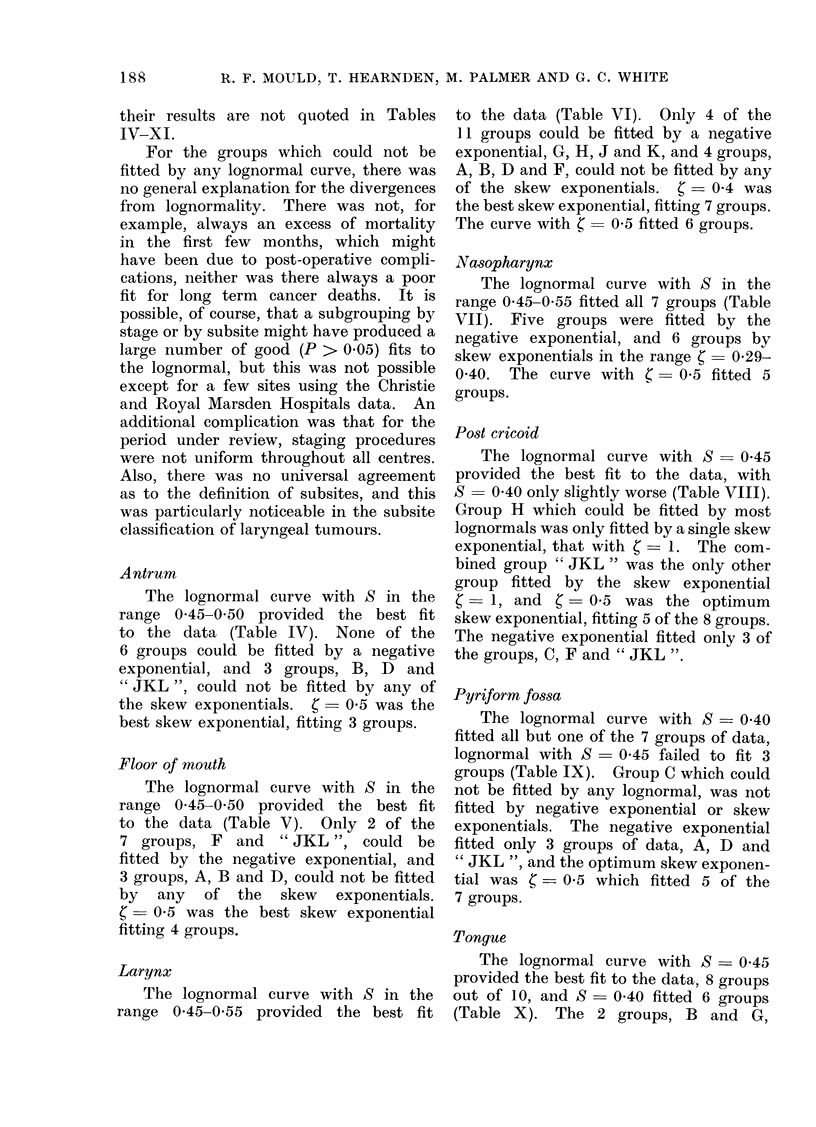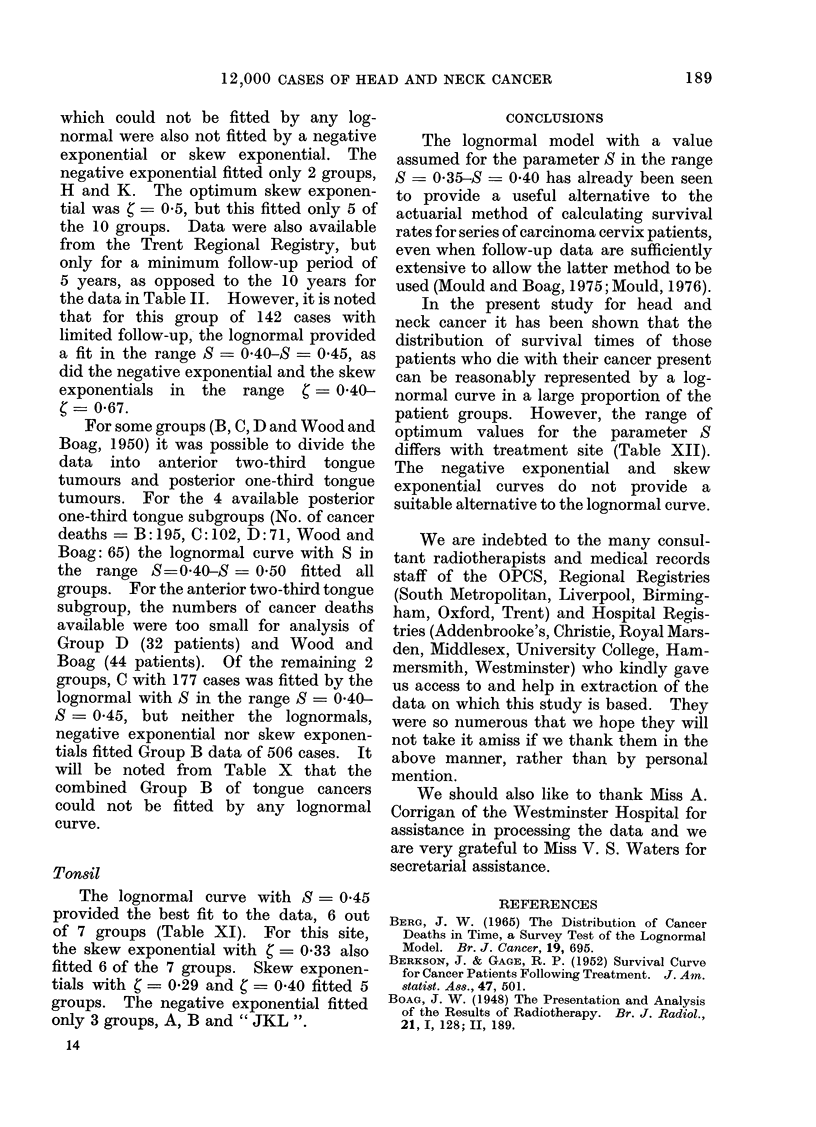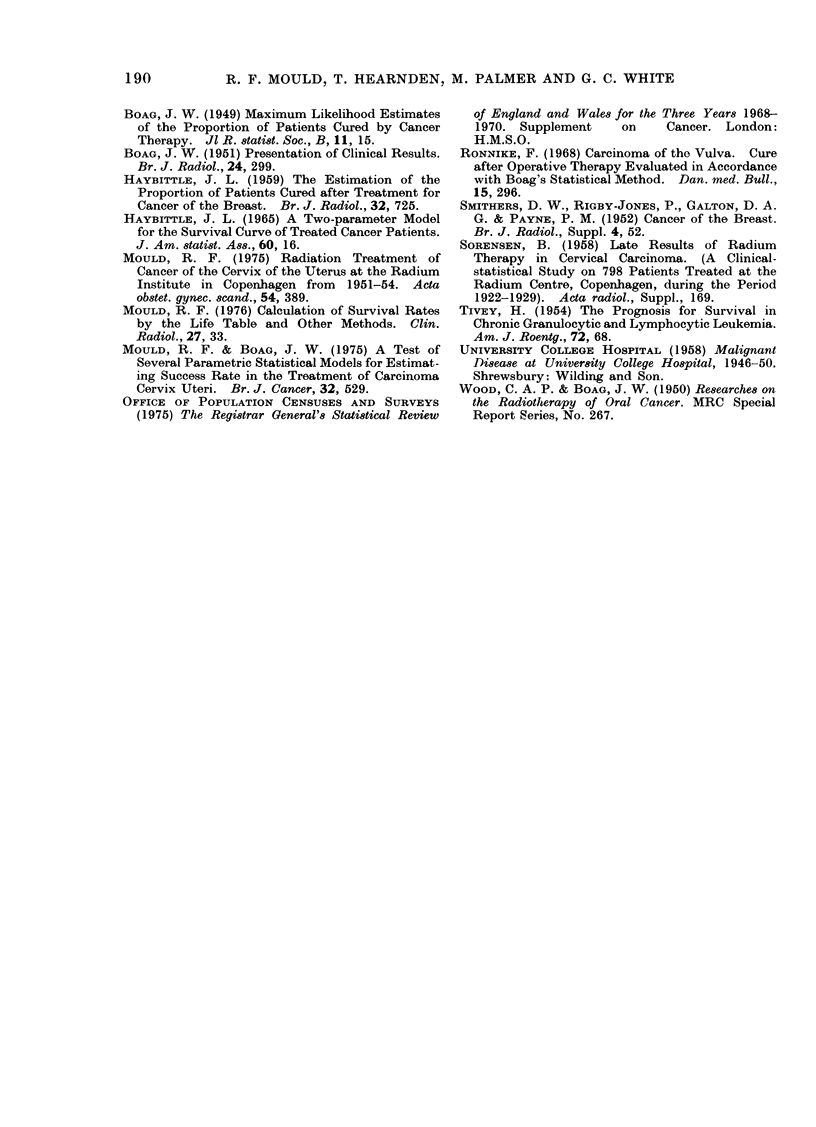# Distribution of survival times of 12,000 head and neck cancer patients who died with their disease.

**DOI:** 10.1038/bjc.1976.141

**Published:** 1976-08

**Authors:** R. F. Mould, T. Hearnden, M. Palmer, G. C. White

## Abstract

The lognormal parametric statistical model can provide, for groups of carcinoma cervix patients, good estimates of long-term survival fractions several years earlier than would otherwise be possible. The present paper extends this model work to head and neck cancer by using a minimum chi-squared test for goodness of fit (P greater than 0-05), to study the distribution of survival times of patients who died with their cancer present. Some 12,000 case histories were available from 7 hospital registries, 4 regional cancer registries and one national registry (the OPCS). All histories were followed up for at least 10 years subsequent to treatment and could be grouped into one of 8 cancer sites: antrum, floor of mouth, larynx, nasopharynx, pyriform fossa, post cricoid, tonsil and tongue. The theoretical distributions investigated were the lognormal, negative exponential and skew exponential. The results showed that the lognormal provided the best overall fit to the data, although the range of optimum values for the lognormal parameter, S, differed with cancer site. The optimum range did, however, usually include the value S=0-45. These results will now permit the second stage of validation of the lognormal model to proceed for head and neck cancers.


*Br. J. Cancer* (1976) **34**, 180

### DISTRIBUTION OF SURVIVAL TIMES OF 12,000 HEAD AND NECK CANCER PATIENTS WHO DIED WITH THEIR DISEASE

R. F. MOULD*[1], T. HEARNDEN[2], M. PALMER[3] AND G. C. WHITE

*From [1]the Westminster Hospital, London SW1, [2]Christie Hospital and Holt Radium Institute, Manchester and [3]the Office of Population Censuses and Surveys, London*

Received 24 March 1976 Accepted 14 April 1976

**Summary.—The lognormal parametric statistical model can provide, for groups of carcinoma cervix patients, good estimates of long-term survival fractions several years earlier than would otherwise be possible. The present paper extends this model work to head and neck cancer by using a minimum chi-squared test for goodness of fit ($P > 0{\cdot}05$), to study the distribution of survival times of patients who died with their cancer present. Some 12,000 case histories were available from 7 hospital registries, 4 regional cancer registries and one national registry (the OPCS). All histories were followed up for at least 10 years subsequent to treatment and could be grouped into one of 8 cancer sites: antrum, floor of mouth, larynx, nasopharynx, pyriform fossa, post cricoid, tonsil and tongue. The theoretical distributions investigated were the lognormal, negative exponential and skew exponential. The results showed that the lognormal provided the best overall fit to the data, although the range of optimum values for the lognormal parameter, S, differed with cancer site. The optimum range did, however, usually include the value S = 0·45. These results will now permit the second stage of validation of the lognormal model to proceed for head and neck cancers.**

THE USE of parametric statistical models to predict the long term survival rates for groups of patients treated for cancer was suggested many years ago, Boag, 1948; Berkson and Gage, 1952; Haybittle, 1959, 1965. Until recently, however, the models could not be adequately tested due to the lack of patient data with sufficiently detailed follow-up, and the lack of the computer facilities necessary for the lengthy iterative procedures. It has been shown that a lognormal model of the type in Fig. 1 is the most consistent model for groups of staged carcinoma cervix patients, when a value is assumed for the lognormal parameter $S$ in the range $S = 0{\cdot}35$–$S = 0{\cdot}40$ (Mould and Boag, 1975).

The validation of this lognormal model for carcinoma cervix, was made in 2 tests. *Firstly*, the actual survival time distribution of each group of patients was compared with the postulated analytical form, choosing the model parameters to give the best fit. The goodness of fit was assessed using a minimum chi-squared test. The optimum analytical form is significant only in that it provides a good *empirical* fit to the data. No *biological* explanation is suggested for the pattern of lognormality. *Secondly*, accepting only the limited survival data which would have been available a few years (2, 3 or 4 years) after the end of a 5-year treatment period under review, the model was used to predict the 10-year or 15-year survival rates. The predicted values were then compared with observed 10-year and 15-year results, taking standard errors into account.

The present paper extends this earlier work (Mould and Boag, 1975) to include

* Correspondence to Dr R. F. Mould, Physics Department, Westminster Hospital, Page Street Wing, London, SW1.

FIG. 1.—Statistical model for cancer of the cervix.

cancers of the head and neck, but only for the *first* of the 2 tests. Data for those who died from an intercurrent disease and those who are still alive has not yet been collected by the authors, but the survival times were available for some 12,000 patients, known to have died with their cancer present, who have all been followed up for at least 10 years subsequent to treatment. All patients had a verified histology prior to initial treatment.

### LITERATURE SURVEY

Lognormality testing has previously been attempted for head and neck cancers by Boag (1948, 1949, 1951), Berg (1965) and Wood and Boag (1950), and for cancer at other sites by Tivey (1954), Sorensen (1958), Smithers *et al.* (1952), Haybittle (1959), Ronnike (1968) and Rabbe (see Mould, 1975). The methods used to test for goodness of fit have varied, and although a $\chi^2$ test using a single set of estimated lognormal parameters has occasionally been used, a thorough minimum $\chi^2$ test such as that which will be described in this paper has never previously been attempted. The head and neck data used by Berg (1965) was that published by University College Hospital (1958) for the treatment period 1946–50, and the numbers of cancer deaths for individual sites were very small. More recent U.C.H. data is used in the present study. The data of Wood and Boag (1950) was also more recently followed up by one of us (R.F.M.) in 1967, and was re-tested for lognormality.

### MATERIALS AND METHODS

To investigate adequately an analytical form for the distribution of cancer death survival times, all patients must have been observed for at least 10 years subsequent to treatment, otherwise, the " tail " of the distribution cannot be verified. Data from the regional registries in England and Wales are collated centrally by the Office of Population Censuses and Surveys (OPCS). Patients are routinely followed up at intervals of 5, 10 and 15 years after the initial registration. Those registrations relating to the period 1954–55 have recently been followed up and 15 year survival rates determined (OPCS, 1975). That part of the OPCS data relevant to head and neck cancer was made available to the authors in the form of individual survival times of patients with proven histology who eventually died with their cancer present. Data were also obtained from various Regional Cancer Registries but only for a minimum follow-up period of 10 years.

Individual hospital cancer registries vary widely in their methods of data collection, storage and retrieval. In the best registries, most treated cases will have been histologically verified and have well-recorded long follow-ups, but there will often be the disadvantage that the numbers of cases for any given treatment site are too small for this type of study. This was overcome by choosing 2 radiotherapy centres with a large intake of patients, both of which have excellent records dating back to the early 1930s. The Christie Hospital, Manchester and the Royal Marsden Hospital, London are probably the only 2 hospital registries in the U.K. with such a wealth of reliable information for head and neck cancer. The 3 London teaching hospitals chosen, Westminster, University College and Middlesex, did not always have sufficient numbers of cases for analysis, and when this occurred, the 3 sets of data were combined. Addenbrooke's Hospital, Cambridge was chosen since work on statistical models had already been carried out at this centre (Haybittle, 1959).

The head and neck anatomical region was chosen for this study since it contains the sites of some cancers with a good prognosis, which require a long term rather than short term survival rate as a criterion for success of treatment. Also, an earlier study had been conducted by Wood and Boag (1950) using Hammersmith Hospital data for the treatment period 1934–45 for several individual head and neck treatment sites, but the number of cases for each site was usually less than 50. In the more recent follow-up of this data, the numbers of cancer deaths increased only slightly.

Eight treatment sites within the head and neck have been chosen for the study (Table I). It was not usually possible to divide the data into staged groups. The Wood and Boag data (1950) contained all the sites in Table I with the exception of pyriform fossa which had been grouped with other sites under the general heading of pharynx. Table II gives a breakdown by registry and treatment site of some 12,000 cases used in this study. Only the Christie Hospital and OPCS data have a minimum follow-up period of 15 years.

*Mathematical formulae.*—Several analytical forms have been proposed as a representation for the death rate ($N(t)$, Fig. 1) of cases who die with cancer present. They have included the lognormal curve (equation 1), the negative exponential (equation 2) and the skew exponential (equation 3).

$$N(t) = \frac{1}{\sqrt{2\pi}}.\exp\left(-\tfrac{1}{2}x^2\right)$$

where

$$x = \frac{\log t - M}{S} \qquad (1)$$

$$N(t) = \alpha.\exp(-\alpha t) \qquad (2)$$

$$N(t) = N_0.t.\exp(-\gamma.t^{\zeta}) \qquad (3)$$

The actual survival time distribution of a group of patients has been compared with the postulated analytical form, choosing the parameters ($M$ and $S$ for the lognormal; $\alpha$ for the negative exponential; $\gamma$ and $\zeta$ for the skew exponential) to give the best fit, and using a computer to assess the goodness of fit by a minimum $\chi^2$ test.

*Minimum $\chi^2$ test*—Agreement between the observed survival time distribution and the proposed formulae was tested by grouping survival times into 4-monthly periods between 0–1 year,* 6-monthly periods between 1–2 years, annual periods between 2–5 years and longer periods thereafter, until a survival time of 10 or 15 years subsequent to treatment was reached. Observed and theoretical numbers in each interval were compared, using a $\chi^2$ test for the data in Table II and for 7 lognormal curves with fixed values of $S$ equal

* For small sample series these groups were sometimes combined, and the initial 2 periods changed to 0–6 months and 6–12 months.

to 0·25, 0·30, 0·35, 0·40, 0·45, 0·50 and 0·55; for the negative exponential and for 7 members of the skew exponential family (equation 3) with $\zeta$ defined by equation (4), where $r$ is integral and $1 \leqslant r \leqslant 7$. This

$$\zeta = 2/(1 + r) \qquad (4)$$

restriction ensured that integration of equation (3) would lead to a complete gamma function which could be easily evaluated. The theoretical parameters ($M$, $\alpha$, $\gamma$) were varied stepwise until a minimum $\chi^2$ was found, and the computer then printed out this value together with the corresponding values of the parameters.

### RESULTS

The data of Wood and Boag (1950) with additional follow-up were tested using the lognormal curves and the results are given in Table III. The number of cancer deaths in each group is too small for any firm conclusions to be drawn, but it does suggest that the lognormal may provide a good fit for most sites, when a value is assumed for the parameter $S$ in the range $S = 0{\cdot}35$–$S = 0{\cdot}45$.

Each site in Table I will be considered individually, since it was found that the optimum range of values for the lognormal parameter $S$ was not the same for all sites. It was noted that although the lognormal with $S = 0{\cdot}25$ was tested, unlike some of the Wood and Boag (1950) data of Table III, none of the data in Groups A–L (Table II) could be fitted by the $S = 0{\cdot}25$ lognormal curve.

TABLE I.—*Treatment Sites*

| |
|---|
| Antrum |
| Floor of mouth |
| Larynx |
| Nasopharynx |
| Post cricoid |
| Pyriform fossa |
| Tongue |
| Tonsil |

TABLE II.—*Grouping by Registry, Treatment Period and Site*

| Registry | Treatment period | Total cases for a given site within the head and neck who died with their cancer present: Antrum | Tongue | Larynx | Nasopharynx | Pyriform fossa | Post cricoid | Floor of mouth | Tonsil | Group reference letter |
|---|---|---|---|---|---|---|---|---|---|---|
| OPCS | 1954–55 | — | 537 | 242 | 194 | 174 | 246 | 179 | 182 | A |
| Christie Hospital, Manchester | 1945–58 | 222 | 700 | 449 | 175 | 142 | 203 | 219 | 139 | B |
| Royal Marsden Hospital, London | 1933–63 | 127 | 366 | 441 | 222 | 268 | 194 | 146 | 80 | C |
| South Metropolitan Regional Registry | 1958–63 | 134 | 290 | 345 | 122 | 155 | 113 | 90 | 157 | D |
| Liverpool Regional Registry | 1951–63 | 98 | 199 | 309 | 105 | 117 | 192 | 99 | 136 | E |
| Birmingham Regional Registry | 1957–63 | 112 | 211 | 310 | 83 | 82 | 78 | 77 | 124 | F |
| Oxford Regional Registry | 1955–63 | — | 90 | 165 | — | — | — | — | — | G |
| Addenbrooke's Hospital, Cambridge | 1945–65 | 44 | 153 | 97 | 31 | 38 | 87 | 43 | 41 | H |
| Middlesex Hospital, London | 1945–65 | 70 | 183 | 143 | 66 | 41 | 29 | 41 | 58 | J |
| University College Hospital, London | 1945–65 | 27 | 77 | 80 | 21 | 42 | 39 | 19 | 27 | K |
| Westminster Hospital, London | 1945–65 | 41 | 33 | 101 | 14 | 28 | 51 | 30 | 22 | L |
| Hammersmith Hospital, London (Wood and Boag, 1950) | 1934–45 | 25 | 103 | 31 | 64* | — | 38 | 32 | 51 | — |
| Total cases for each site | | 900 | 2942 | 2713 | 1097 | 1087 | 1270 | 975 | 1017 | |

*1934–57

The results refer to 63 different groups of patients with between 6 and 11 groups per site (Tables III–XII). The number of cancer deaths/group was in the range 77–700, and one-third of the groups contained more than 180 patients each. In general, for the smaller groups, log-normal distributions with several values of $S$ were found to give adequate fits, whereas for the larger groups fewer fitted the data. Nevertheless, for 6 of the 8 groups containing more than 300 patients at least one value of $S$ was found which gave an adequate fit ($P > 0{\cdot}05$) to a

TABLE III.—*Goodness of Fit of the Wood and Boag* (1950) *Data to the Lognormals*

| Treatment site | No. of cancer deaths | P levels for different values of the lognormal parameter S. Notation: ⊕ signifies $P>0{\cdot}05$; — signifies $P<0{\cdot}05$ | | | | | | |
|---|---|---|---|---|---|---|---|---|
| | | $S=0{\cdot}25$ | $S=0{\cdot}30$ | $S=0{\cdot}35$ | $S=0{\cdot}40$ | $S=0{\cdot}45$ | $S=0{\cdot}50$ | $S=0{\cdot}55$ |
| Antrum | 25 | — | ⊕ | ⊕ | ⊕ | ⊕ | ⊕ | ⊕ |
| Floor of mouth | 32 | ⊕ | ⊕ | ⊕ | ⊕ | ⊕ | ⊕ | — |
| Larynx | 31 | ⊕ | ⊕ | ⊕ | ⊕ | — | — | — |
| Nasopharynx | 64 | — | — | ⊕ | ⊕ | ⊕ | ⊕ | ⊕ |
| Tongue | 103 | — | — | ⊕ | ⊕ | ⊕ | — | — |
| Tonsil | 51 | — | — | ⊕ | ⊕ | ⊕ | ⊕ | ⊕ |
| Post cricoid | 38 | — | — | — | ⊕ | ⊕ | — | — |
| No. of series for which a good fit to the data is obtained ($P>0{\cdot}05$) | | 2 | 3 | 6 | 7 | 6 | 4 | 3 |

In each case the symbol (⊕ or —) in the table gives the result for a minimum goodness of fit test, for the data on that horizontal level and the lognormal curve at the head of the vertical column.

TABLE IV.—*Goodness of Fit of Antrum Data to the Lognormals*

| No. of cancer deaths | Group reference letter (see Table II) | P levels for different values of the lognormal parameter S | | | | | |
|---|---|---|---|---|---|---|---|
| | | $S=0{\cdot}30$ | $S=0{\cdot}35$ | $S=0{\cdot}40$ | $S=0{\cdot}45$ | $S=0{\cdot}50$ | $S=0{\cdot}55$ |
| 127 | C | — | ⊕ | ⊕ | ⊕ | ⊕ | — |
| 112 | F | — | ⊕ | ⊕ | ⊕ | ⊕ | — |
| 138 | JKL | — | — | — | ⊕ | ⊕ | — |
| 98 | E | — | — | — | ⊕ | ⊕ | ⊕ |
| 134 | D | — | — | — | ⊕ | ⊕ | ⊕ |
| 222 | B | — | — | — | — | — | — |
| Number of series out of 6 for which a good fit to the data is obtained ($P>0{\cdot}05$) | | — | 2 | 2 | 5 | 5 | 2 |

Symbols as in Table III.

TABLE V.—*Goodness of Fit of Floor of Mouth Data to the Lognormals*

| No. of cancer deaths | Group reference letter (see Table II) | P levels for different values of the lognormal parameter S | | | | | |
|---|---|---|---|---|---|---|---|
| | | $S=0{\cdot}30$ | $S=0{\cdot}35$ | $S=0{\cdot}40$ | $S=0{\cdot}45$ | $S=0{\cdot}50$ | $S=0{\cdot}55$ |
| 77 | F | — | ⊕ | ⊕ | ⊕ | ⊕ | ⊕ |
| 90 | JKL | — | ⊕ | ⊕ | ⊕ | ⊕ | ⊕ |
| 146 | C | — | — | ⊕ | ⊕ | ⊕ | ⊕ |
| 99 | E | — | — | ⊕ | ⊕ | ⊕ | — |
| 90 | D | — | — | — | ⊕ | ⊕ | ⊕ |
| 179 | A | — | — | — | — | — | — |
| 219 | B | — | — | — | — | — | — |
| Number of series out of 7 for which a good fit to the data is obtained ($P>0{\cdot}05$) | | — | 2 | 4 | 5 | 5 | 4 |

Symbols as in Table III.

TABLE VI.—*Goodness of Fit of Larynx Data to the Lognormals*

| No. of cancer deaths | Group reference letter (see Table II) | *P* levels for different values of the lognormal parameter *S* | | | | | |
|---|---|---|---|---|---|---|---|
| | | $S=0\cdot30$ | $S=0\cdot35$ | $S=0\cdot40$ | $S=0\cdot45$ | $S=0\cdot50$ | $S=0\cdot55$ |
| 97 | H | — | — | ⊕ | ⊕ | ⊕ | ⊕ |
| 80 | K | — | — | — | ⊕ | ⊕ | ⊕ |
| 143 | J | — | — | ⊕ | ⊕ | ⊕ | ⊕ |
| 101 | L | — | — | ⊕ | ⊕ | ⊕ | ⊕ |
| 449 | B | — | — | — | ⊕ | ⊕ | ⊕ |
| 441 | C | — | — | — | ⊕ | ⊕ | ⊕ |
| 165 | G | — | — | — | ⊕ | ⊕ | ⊕ |
| 309 | E | — | — | — | ⊕ | ⊕ | ⊕ |
| 310 | F | — | — | — | ⊕ | ⊕ | — |
| 242 | A | — | — | — | — | — | ⊕ |
| 345 | D | — | — | — | — | — | — |
| Number of series out of 11 for which a good fit to the data is obtained ($P > 0\cdot05$) | | — | — | 3 | 9 | 9 | 9 |

Symbols as in Table III.

TABLE VII.—*Goodness of Fit of Nasopharynx Data to the Lognormals*

| No. of cancer deaths | Group reference letter (see Table II) | *P* levels for different values of the lognormal parameter *S* | | | | | |
|---|---|---|---|---|---|---|---|
| | | $S=0\cdot30$ | $S=0\cdot35$ | $S=0\cdot40$ | $S=0\cdot45$ | $S=0\cdot50$ | $S=0\cdot55$ |
| 101 | JKL | — | ⊕ | ⊕ | ⊕ | ⊕ | ⊕ |
| 175 | B | — | — | ⊕ | ⊕ | ⊕ | ⊕ |
| 83 | F | — | — | ⊕ | ⊕ | ⊕ | ⊕ |
| 194 | A | — | — | — | ⊕ | ⊕ | ⊕ |
| 222 | C | — | — | — | ⊕ | ⊕ | ⊕ |
| 122 | D | — | — | ⊕ | ⊕ | ⊕ | ⊕ |
| 105 | E | — | — | ⊕ | ⊕ | ⊕ | ⊕ |
| Number of series out of 7 for which a good fit to the data is obtained ($P > 0\cdot05$) | | — | 1 | 5 | 7 | 7 | 7 |

Symbols as in Table III.

TABLE VIII.—*Goodness of Fit of Post Cricoid Data to the Lognormals*

| No. of cancer deaths | Group reference letter (see Table II) | *P* levels for different values of the lognormal parameter *S* | | | | | |
|---|---|---|---|---|---|---|---|
| | | $S=0\cdot30$ | $S=0\cdot35$ | $S=0\cdot40$ | $S=0\cdot45$ | $S=0\cdot50$ | $S=0\cdot55$ |
| 119 | JKL | ⊕ | ⊕ | ⊕ | ⊕ | — | — |
| 78 | F | — | ⊕ | ⊕ | ⊕ | ⊕ | ⊕ |
| 203 | B | — | ⊕ | ⊕ | ⊕ | — | — |
| 194 | C | — | — | ⊕ | ⊕ | ⊕ | ⊕ |
| 113 | D | — | — | ⊕ | — | — | — |
| 246 | A | — | — | — | ⊕ | ⊕ | ⊕ |
| 192 | E | — | — | — | ⊕ | ⊕ | — |
| 87 | H | ⊕ | ⊕ | ⊕ | ⊕ | ⊕ | — |
| Number of series out of 8 for which a good fit to the data is obtained ($P > 0\cdot05$) | | 2 | 4 | 6 | 7 | 5 | 3 |

Symbols as in Table III.

TABLE IX.—*Goodness of Fit of Pyriform Fossa data to the Lognormals*

| No. of cancer deaths | Group reference letter (see Table II) | P levels for different values of the lognormal parameter S | | | | | |
|---|---|---|---|---|---|---|---|
| | | $S=0\cdot30$ | $S=0\cdot35$ | $S=0\cdot40$ | $S=0\cdot45$ | $S=0\cdot50$ | $S=0\cdot55$ |
| 142 | B | ⊕ | ⊕ | ⊕ | — | — | — |
| 111 | JKL | — | ⊕ | ⊕ | ⊕ | ⊕ | — |
| 155 | D | — | ⊕ | ⊕ | ⊕ | ⊕ | ⊕ |
| 174 | A | — | — | ⊕ | ⊕ | ⊕ | ⊕ |
| 82 | F | — | — | ⊕ | ⊕ | — | — |
| 117 | E | — | — | ⊕ | — | — | — |
| 268 | C | — | — | — | — | — | — |
| Number of series out of 7 for which a good fit to the data is obtained ($P > 0\cdot05$) | | 1 | 3 | 6 | 4 | 3 | 2 |

Symbols as in Table III.

TABLE X.—*Goodness of Fit of Tongue Data to the Lognormals*

| No. of cancer deaths | Group reference letter (see Table II) | P levels for different values of the lognormal parameter S | | | | | |
|---|---|---|---|---|---|---|---|
| | | $S=0\cdot30$ | $S=0\cdot35$ | $S=0\cdot40$ | $S=0\cdot45$ | $S=0\cdot50$ | $S=0\cdot55$ |
| 77 | K | ⊕ | ⊕ | ⊕ | ⊕ | — | — |
| 153 | H | — | ⊕ | ⊕ | ⊕ | — | — |
| 199 | E | — | — | ⊕ | ⊕ | ⊕ | ⊕ |
| 211 | F | — | — | ⊕ | ⊕ | ⊕ | ⊕ |
| 183 | J | — | — | ⊕ | ⊕ | ⊕ | — |
| 366 | C | — | — | ⊕ | ⊕ | — | — |
| 537 | A | — | — | — | ⊕ | — | — |
| 290 | D | — | — | — | ⊕ | — | — |
| 700 | B | — | — | — | — | — | — |
| 90 | G | — | — | — | — | — | — |
| Number of series out of 10 for which a good fit to the data is obtained ($P > 0\cdot05$) | | 1 | 2 | 6 | 8 | 3 | 2 |

Symbols as in Table III.

TABLE XI.—*Goodness of Fit of Tonsil Data to the Lognormals*

| No. of cancer deaths | Group reference letter (see Table II) | P levels for different values of the lognormal parameter S | | | | | |
|---|---|---|---|---|---|---|---|
| | | $S=0\cdot30$ | $S=0\cdot35$ | $S=0\cdot40$ | $S=0\cdot45$ | $S=0\cdot50$ | $S=0\cdot55$ |
| 139 | B | — | — | ⊕ | ⊕ | ⊕ | ⊕ |
| 182 | A | — | — | — | ⊕ | ⊕ | ⊕ |
| 157 | D | — | — | — | ⊕ | ⊕ | ⊕ |
| 136 | E | — | — | — | ⊕ | ⊕ | ⊕ |
| 107 | JKL | — | — | — | ⊕ | ⊕ | — |
| 124 | F | — | — | — | — | — | ⊕ |
| 80 | C | — | — | — | ⊕ | — | — |
| Number of series out of 7 for which a good fit to the data is obtained ($P > 0\cdot05$) | | — | — | 1 | 6 | 5 | 5 |

Symbols as in Table III.

lognormal distribution. Fig. 2 shows 2 examples of survival time distributions fitted by lognormal curves.

It was found that for all 8 sites, at least 2 lognormal curves fitted more groups of observational data than did the negative exponential curve. In comparison with the skew exponential curves, the lognormal fitted at least 2 more data groups for antrum, floor of mouth, larynx and tongue. For nasopharynx, post cricoid and pyriform fossa, however, the

TABLE XII.—*Goodness of Fit of all Data Groups to the Lognormals*

| Cancer site | Total no. of groups tested | No. of groups for which a good fit to the data is obtained ($P > 0{\cdot}05$), for lognormal curves with different values assumed for $S$ | | | | | |
|---|---|---|---|---|---|---|---|
| | | $S = 0{\cdot}30$ | $S = 0{\cdot}35$ | $S = 0{\cdot}40$ | $S = 0{\cdot}45$ | $S = 0{\cdot}50$ | $S = 0{\cdot}55$ |
| Antrum | 6 | — | 2 | 2 | 5 | 5 | 2 |
| Floor of mouth | 7 | — | 2 | 4 | 5 | 5 | 4 |
| Larynx | 11 | — | — | 3 | 9 | 9 | 9 |
| Nasopharynx | 7 | — | 1 | 5 | 7 | 7 | 7 |
| Post cricoid | 8 | 2 | 4 | 6 | 7 | 5 | 3 |
| Pyriform fossa | 7 | 1 | 3 | 6 | 4 | 3 | 2 |
| Tongue | 10 | 1 | 2 | 6 | 8 | 3 | 2 |
| Tonsil | 7 | — | — | 1 | 6 | 5 | 5 |

lognormal fitted only one more data group than the skew exponential curve. Only for a single site, the tonsil, did one skew exponential curve, $\zeta = 0{\cdot}33$, fit as many groups of data as a lognormal curve. Thus, since negative exponential and skew exponential curves offer only a poor alternative to lognormal curves,

FIG. 2.—Distribution of survival times of patients who died with cancer present: histogram of observations and theoretical lognormal curves.

their results are not quoted in Tables IV–XI.

For the groups which could not be fitted by any lognormal curve, there was no general explanation for the divergences from lognormality. There was not, for example, always an excess of mortality in the first few months, which might have been due to post-operative complications, neither was there always a poor fit for long term cancer deaths. It is possible, of course, that a subgrouping by stage or by subsite might have produced a large number of good ($P > 0{\cdot}05$) fits to the lognormal, but this was not possible except for a few sites using the Christie and Royal Marsden Hospitals data. An additional complication was that for the period under review, staging procedures were not uniform throughout all centres. Also, there was no universal agreement as to the definition of subsites, and this was particularly noticeable in the subsite classification of laryngeal tumours.

*Antrum*

The lognormal curve with $S$ in the range 0·45–0·50 provided the best fit to the data (Table IV). None of the 6 groups could be fitted by a negative exponential, and 3 groups, B, D and " JKL ", could not be fitted by any of the skew exponentials. $\zeta = 0{\cdot}5$ was the best skew exponential, fitting 3 groups.

*Floor of mouth*

The lognormal curve with $S$ in the range 0·45–0·50 provided the best fit to the data (Table V). Only 2 of the 7 groups, F and " JKL ", could be fitted by the negative exponential, and 3 groups, A, B and D, could not be fitted by any of the skew exponentials. $\zeta = 0{\cdot}5$ was the best skew exponential fitting 4 groups.

*Larynx*

The lognormal curve with $S$ in the range 0·45–0·55 provided the best fit to the data (Table VI). Only 4 of the 11 groups could be fitted by a negative exponential, G, H, J and K, and 4 groups, A, B, D and F, could not be fitted by any of the skew exponentials. $\zeta = 0{\cdot}4$ was the best skew exponential, fitting 7 groups. The curve with $\zeta = 0{\cdot}5$ fitted 6 groups.

*Nasopharynx*

The lognormal curve with $S$ in the range 0·45–0·55 fitted all 7 groups (Table VII). Five groups were fitted by the negative exponential, and 6 groups by skew exponentials in the range $\zeta = 0{\cdot}29$–0·40. The curve with $\zeta = 0{\cdot}5$ fitted 5 groups.

*Post cricoid*

The lognormal curve with $S = 0{\cdot}45$ provided the best fit to the data, with $S = 0{\cdot}40$ only slightly worse (Table VIII). Group H which could be fitted by most lognormals was only fitted by a single skew exponential, that with $\zeta = 1$. The combined group " JKL " was the only other group fitted by the skew exponential $\zeta = 1$, and $\zeta = 0{\cdot}5$ was the optimum skew exponential, fitting 5 of the 8 groups. The negative exponential fitted only 3 of the groups, C, F and " JKL ".

*Pyriform fossa*

The lognormal curve with $S = 0{\cdot}40$ fitted all but one of the 7 groups of data, lognormal with $S = 0{\cdot}45$ failed to fit 3 groups (Table IX). Group C which could not be fitted by any lognormal, was not fitted by negative exponential or skew exponentials. The negative exponential fitted only 3 groups of data, A, D and " JKL ", and the optimum skew exponential was $\zeta = 0{\cdot}5$ which fitted 5 of the 7 groups.

*Tongue*

The lognormal curve with $S = 0{\cdot}45$ provided the best fit to the data, 8 groups out of 10, and $S = 0{\cdot}40$ fitted 6 groups (Table X). The 2 groups, B and G, which could not be fitted by any lognormal were also not fitted by a negative exponential or skew exponential. The negative exponential fitted only 2 groups, H and K. The optimum skew exponential was $\zeta = 0{\cdot}5$, but this fitted only 5 of the 10 groups. Data were also available from the Trent Regional Registry, but only for a minimum follow-up period of 5 years, as opposed to the 10 years for the data in Table II. However, it is noted that for this group of 142 cases with limited follow-up, the lognormal provided a fit in the range $S = 0{\cdot}40$–$S = 0{\cdot}45$, as did the negative exponential and the skew exponentials in the range $\zeta = 0{\cdot}40$–$\zeta = 0{\cdot}67$.

For some groups (B, C, D and Wood and Boag, 1950) it was possible to divide the data into anterior two-third tongue tumours and posterior one-third tongue tumours. For the 4 available posterior one-third tongue subgroups (No. of cancer deaths = B: 195, C: 102, D: 71, Wood and Boag: 65) the lognormal curve with S in the range $S=0{\cdot}40$–$S = 0{\cdot}50$ fitted all groups. For the anterior two-third tongue subgroup, the numbers of cancer deaths available were too small for analysis of Group D (32 patients) and Wood and Boag (44 patients). Of the remaining 2 groups, C with 177 cases was fitted by the lognormal with $S$ in the range $S = 0{\cdot}40$–$S = 0{\cdot}45$, but neither the lognormals, negative exponential nor skew exponentials fitted Group B data of 506 cases. It will be noted from Table X that the combined Group B of tongue cancers could not be fitted by any lognormal curve.

### *Tonsil*

The lognormal curve with $S = 0{\cdot}45$ provided the best fit to the data, 6 out of 7 groups (Table XI). For this site, the skew exponential with $\zeta = 0{\cdot}33$ also fitted 6 of the 7 groups. Skew exponentials with $\zeta = 0{\cdot}29$ and $\zeta = 0{\cdot}40$ fitted 5 groups. The negative exponential fitted only 3 groups, A, B and " JKL ".

### CONCLUSIONS

The lognormal model with a value assumed for the parameter $S$ in the range $S = 0{\cdot}35$–$S = 0{\cdot}40$ has already been seen to provide a useful alternative to the actuarial method of calculating survival rates for series of carcinoma cervix patients, even when follow-up data are sufficiently extensive to allow the latter method to be used (Mould and Boag, 1975; Mould, 1976).

In the present study for head and neck cancer it has been shown that the distribution of survival times of those patients who die with their cancer present can be reasonably represented by a lognormal curve in a large proportion of the patient groups. However, the range of optimum values for the parameter $S$ differs with treatment site (Table XII). The negative exponential and skew exponential curves do not provide a suitable alternative to the lognormal curve.

We are indebted to the many consultant radiotherapists and medical records staff of the OPCS, Regional Registries (South Metropolitan, Liverpool, Birmingham, Oxford, Trent) and Hospital Registries (Addenbrooke's, Christie, Royal Marsden, Middlesex, University College, Hammersmith, Westminster) who kindly gave us access to and help in extraction of the data on which this study is based. They were so numerous that we hope they will not take it amiss if we thank them in the above manner, rather than by personal mention.

We should also like to thank Miss A. Corrigan of the Westminster Hospital for assistance in processing the data and we are very grateful to Miss V. S. Waters for secretarial assistance.

### REFERENCES

Berg, J. W. (1965) The Distribution of Cancer Deaths in Time, a Survey Test of the Lognormal Model. *Br. J. Cancer*, **19**, 695.

Berkson, J. & Gage, R. P. (1952) Survival Curve for Cancer Patients Following Treatment. *J. Am. statist. Ass.*, **47**, 501.

Boag, J. W. (1948) The Presentation and Analysis of the Results of Radiotherapy. *Br. J. Radiol.*, **21**, I, 128; II, 189.

BOAG, J. W. (1949) Maximum Likelihood Estimates of the Proportion of Patients Cured by Cancer Therapy. *Jl R. statist. Soc., B*, **11**, 15.

BOAG, J. W. (1951) Presentation of Clinical Results. *Br. J. Radiol.*, **24**, 299.

HAYBITTLE, J. L. (1959) The Estimation of the Proportion of Patients Cured after Treatment for Cancer of the Breast. *Br. J. Radiol.*, **32**, 725.

HAYBITTLE, J. L. (1965) A Two-parameter Model for the Survival Curve of Treated Cancer Patients. *J. Am. statist. Ass.*, **60**, 16.

MOULD, R. F. (1975) Radiation Treatment of Cancer of the Cervix of the Uterus at the Radium Institute in Copenhagen from 1951–54. *Acta obstet. gynec. scand.*, **54**, 389.

MOULD, R. F. (1976) Calculation of Survival Rates by the Life Table and Other Methods. *Clin. Radiol.*, **27**, 33.

MOULD, R. F. & BOAG, J. W. (1975) A Test of Several Parametric Statistical Models for Estimating Success Rate in the Treatment of Carcinoma Cervix Uteri. *Br. J. Cancer*, **32**, 529.

OFFICE OF POPULATION CENSUSES AND SURVEYS (1975) *The Registrar General's Statistical Review of England and Wales for the Three Years* 1968–1970. Supplement on Cancer. London: H.M.S.O.

RONNIKE, F. (1968) Carcinoma of the Vulva. Cure after Operative Therapy Evaluated in Accordance with Boag's Statistical Method. *Dan. med. Bull.*, **15**, 296.

SMITHERS, D. W., RIGBY-JONES, P., GALTON, D. A. G. & PAYNE, P. M. (1952) Cancer of the Breast. *Br. J. Radiol.*, Suppl. **4**, 52.

SORENSEN, B. (1958) Late Results of Radium Therapy in Cervical Carcinoma. (A Clinical-statistical Study on 798 Patients Treated at the Radium Centre, Copenhagen, during the Period 1922–1929). *Acta radiol.*, Suppl., 169.

TIVEY, H. (1954) The Prognosis for Survival in Chronic Granulocytic and Lymphocytic Leukemia. *Am. J. Roentg.*, **72**, 68.

UNIVERSITY COLLEGE HOSPITAL (1958) *Malignant Disease at University College Hospital*, 1946–50. Shrewsbury: Wilding and Son.

WOOD, C. A. P. & BOAG, J. W. (1950) *Researches on the Radiotherapy of Oral Cancer*. MRC Special Report Series, No. 267.